# Perceptions of clinicians and staff about the use of digital technology in primary care: qualitative interviews prior to implementation of a computer-facilitated 5As intervention

**DOI:** 10.1186/s12911-016-0284-5

**Published:** 2016-04-19

**Authors:** Anna María Nápoles, Nicole Appelle, Sara Kalkhoran, Maya Vijayaraghavan, Nicholas Alvarado, Jason Satterfield

**Affiliations:** Division of General Internal Medicine, Department of Medicine, University of California San Francisco (UCSF), Box 0856, 3333 California Street, Suite 335, San Francisco, CA 94118 USA; Division of General Internal Medicine, Department of Medicine, UCSF, Box 0320, 1545 Divisadero St., San Francisco, CA 94115 USA; Division of General Internal Medicine, Department of Medicine, Massachusetts General Hospital, 50 Staniford St, 9th Floor, Boston, MA 02114 USA; UCSF, Box 1364, 1001 Potrero Ave., San Francisco General Hospital 90, Room 1311E, San Francisco, USA; Division of General Internal Medicine, Department of Medicine, UCSF, Box 0320, 2200 Post St., MZ Bldg C Room C126B, San Francisco, CA 94115 USA; Division of General Internal Medicine, Department of Medicine, UCSF, Box 1731, 1701 Divisadero St., Room 500, San Francisco, CA 94115 USA

**Keywords:** Smoking cessation counseling, 5As, Computer technology, Primary care, Tobacco addiction

## Abstract

**Background:**

Digital health interventions using hybrid delivery models may offer efficient alternatives to traditional behavioral counseling by addressing obstacles of time, resources, and knowledge. Using a computer-facilitated 5As (ask, advise, assess, assist, arrange) model as an example (CF5As), we aimed to identify factors from the perspectives of primary care providers and clinical staff that were likely to influence introduction of digital technology and a CF5As smoking cessation counseling intervention. In the CF5As model, patients self-administer a tablet intervention that provides 5As smoking cessation counseling, produces patient and provider handouts recommending next steps, and is followed by a patient-provider encounter to reinforce key cessation messages, provide assistance, and arrange follow-up.

**Methods:**

Semi-structured in-person interviews of administrative and clinical staff and primary care providers from three primary care clinics.

**Results:**

Thirty-five interviews were completed (12 administrative staff, ten clinical staff, and 13 primary care providers). Twelve were from an academic internal medicine practice, 12 from a public hospital academic general medicine clinic, and 11 from a public hospital HIV clinic. Most were women (91 %); mean age (SD) was 42 years (11.1). Perceived usefulness of the CF5As focused on its relevance for various health behavior counseling purposes, potential gains in counseling efficiency, confidentiality of data collection, occupying patients while waiting, and serving as a cue to action. Perceived ease of use was viewed to depend on the ability to accommodate: clinic workflow; heavy patient volumes; and patient characterisitics, e.g., low literacy. Social norms potentially affecting implementation included beliefs in the promise/burden of technology, priority of smoking cessation counseling relative to other patient needs, and perception of CF5As as just “one more thing to do” in an overburdened system. The most frequently cited facilitating conditions were staffing levels and smoking cessation resources and training; the most cited hindering factors were visit time constraints and patients’ complex health care needs.

**Conclusions:**

Integrating CF5As and other technology-enhanced behavioral counseling interventions in primary care requires flexibility to accommodate work flow and perceptions of overload in dynamic environments. Identifying factors that promote and hinder CF5As adoption could inform implementation of other CF behavioral health interventions in primary care.

## Background

National health care reform in the United States is creating an unprecedented influence on the ways in which health care systems deliver behavioral counseling services [1, 2]. The Health Information Technology for Economic and Clinical Health Act (HITECH Act) was enacted under Title XIII of the American Recovery and Reinvestment Act of 2009 to promote the adoption and meaningful use of health information technology (including electronic health records or EHR) to integrate and improve the quality, efficiency, and safety of care, including preventive and screening services [3]. As a part of the HITECH Act, substantial federal financial incentives are tied to the ability of health care systems to demonstrate compliance with a number of indicators of uses of each provider’s or system’s EHR to improve the quality of care (meaningful use criteria). The purpose of these incentives is to accelerate the adoption of EHR systems among providers. Additionally, provisions of the Patient Protection and Affordable Care Act 2010 expand access to behavioral health for millions of Americans and call for better integration of primary care and behavioral health services [2]. As a result of these two major policy initiatives and increasing opportunities offered by some new technologies to support health care, dynamic changes are occurring in the tools and systems used to screen for and deliver behavioral counseling services in primary care.

In this changing environment, digital health interventions, and the innovative hybrid service delivery models they make possible, may offer efficient alternatives to traditional behavioral counseling and address common obstacles of time, resources, clinician competence, and integration with clinic workflow and EHR [4–6]. In particular, mobile smartphone and tablet applications with linked clinician “dashboards” offer the possibility of “extending” the clinician’s effect beyond the clinic visit. Such models introduce the potential for more frequent, real world data collection that dynamically adjusts an intervention to the evolving needs of patients, while providing important evidence-based decision support to the provider or clinical team [7]. Unfortunately, adherence to mobile app or tablet usage is poor and clinics often fail to fully implement or sustain technological innovations [8]. A better understanding of staff, provider, and patient attitudes and beliefs regarding the use of digital tools for behavioral counseling and other facilitating contextual factors could provide essential insights into the design and implementation of technologies that are effective, efficient, and sustainable [9].

The U.S. Preventive Services Task Force (USPSTF) recommendations to prevent tobacco use and tobacco-related disease in adults call on physicians to identify patients who use tobacco, and provide counseling and pharmacological treatments [10]. Consistent use of the 5As (ask every patient at every visit about smoking, advise users to quit, assess readiness to quit, assist with counseling and pharmacotherapy strategies, and arrange follow-up visit to discuss further) to promote smoking cessation in primary care could significantly reduce tobacco-related morbidity and mortality, [11] but routine adoption has been lacking [12]. In a 2008 survey, primary care providers reported routinely asking nearly all of their patients about tobacco use, advising them to quit, and assisting them in making a quit attempt; however only half reported arranging a follow-up visit [13]. Patient-reported rates of physician counseling are much lower. An analysis of 2001–2010 National Health Interview Survey data found that 48 % of patients had been advised to quit and 32 % had used counseling and/or medications in quit attempts [14]. Clinicians play a critical role in these efforts with evidence indicating that even brief counseling is effective, with effectiveness increasing commensurate with the intensity of counseling [12, 15]. These missed opportunities for providing tobacco cessation counseling are optimal intervention points.

Primary care clinicians cite competing health priorities, lack of self-efficacy and resources, and lack of patient readiness as the most common barriers to the provision of smoking cessation counseling [16]. Telephone quit lines and web-based cessation programs have the advantage of reducing the counseling burden of primary care providers and allowing patients flexibility and control. Clinician-delivered counseling can significantly augment other strategies for smoking cessation, such as self-help and telephone counseling interventions [17–19]. However, physicians may not be aware of these patient-initiated cessation efforts, preventing their integration with primary care efforts to reinforce quit attempts.

To address physician barriers and promote patient engagement in clinic-based cessation efforts, we developed a computer-facilitated 5As (CF5As) service delivery model to test its effect on 5As implementation in primary care. In the CF5As model, patients self-administer a tablet intervention, which asks about smoking, advises users to quit, assesses readiness to quit, and introduces counseling and pharmacotherapy strategies for cessation. The tablet then produces patient and provider handouts recommending next steps, and is followed by a patient-provider encounter to reinforce key cessation messages, provide assistance with cessation, and arrange follow-up. This model begins with a patient self-administered computer tablet intervention (5As) in the primary care waiting room that collects and integrates patient readiness information, produces separate handouts for the patient and provider delineating recommended next steps, and ends with a patient-provider exchange to reinforce key cessation messages, provide additional cessation assistance, and arrange follow-up.

This article describes the results of semi-structured interviews with administrative staff, clinical staff, and primary care clinicians conducted prior to implementation of the CF5As in which the Technology Acceptance Model (TAM) was applied to identify multi-level factors that could affect implementation of technology in general, and the CF5As, in particular, in primary care clinics [20]. According to the TAM, four key constructs predict technology adoption: perceived usefulness (degree to which health technology enhances job performance), ease of use (effort required to use health technology), social norms (influence of group beliefs or behaviors on individual beliefs or use of health technology), and facilitating conditions (beliefs regarding infrastructure, resource constraints, skills, and opportunities to use health technology) [20–22]. Based on the TAM, we posed the following research questions: What do clinic personnel perceive about the usefulness and ease of use of the CF5As in primary care clinics? What beliefs, attitudes and social norms are held by clinic personnel with respect to smoking cessation counseling and use of technology for behavioral counseling? What are the perceived facilitating conditions that could affect implementation of CF5As?

## Methods

### Participant recruitment

A purposive sample was recruited in the San Francisco Bay Area from three primary care clinics participating in a larger implementation study on the use of a computer tablet-assisted 5As smoking cessation intervention [23]. The three sites were an academic internal medicine practice, an academic general medicine practice at a safety-net community hospital, and an academic HIV primary care practice at a safety-net community hospital. The first two sites were selected as the two largest adult primary care clinics at the academic health center, representing a range of diversity of patients and settings. A primary care HIV clinic was chosen as the third site given its high smoking prevalence and emerging recognition of the need for smoking cessation counseling in patients with HIV/AIDS. These sites were selected because they allowed for evaluation of implementation factors across diverse patient populations and clinical settings.

Inclusion criteria were adults who were employees of one of the three clinic sites in which the intervention was to be implemented. Emphasis was placed on selecting and recruiting an equal mix of administrative staff (e.g., patient registration staff), clinical staff (e.g., medical assistants), and primary care providers (including physician assistants and nurse practitioners) because beliefs about the use of technology in primary care could differ by position. Potential participants were sent an initial invitation to participate in the interview via email from the physician investigator from that clinic who was on the research team. The interviewer then followed up via email and telephone to schedule appointments.

### Semi-structured interviews

One-time, individual in-person semi-structured interviews were used to collect information on clinic personnel’s views of the use of computer tablet technology in primary care clinics in general, and more specifically for collecting patient data and providing counseling related to health behaviors, particularly smoking. Using an open-ended script, participants were asked questions about their general attitudes toward tablets and behavioral counseling, their perceptions of the usefulness of tablets, the ease of use of tablets in primary care clinic settings, and social norms in their clinics related to smoking cessation counseling, work flow issues, and the use of technology to aid in behavioral counseling of primary care patients. The script that was developed by the research team identified the open-ended questions that were relevant to the TAM constructs as they might apply in the context of technology implementation in primary care. The script was not pretested. The script started with an ice breaker question that asked respondents to describe their role in the clinic, specified open-ended questions and probes related to each of the TAM constructs (facilitating conditions, perceived usefulness of tablets, perceived ease of use, and social norms), and ended with asking for their suggestions for implementation of tablet technology in primary care practices. The CF5As tablet and its functions were described to participants. The tablet could not be shown to them because it was not available at the time of the interviews.

A female, research professor with a Ph.D., experienced in qualitative research (AMN), conducted the individual 30-minute in-person interviews in private locations at the clinics with no one else present (other than the interviewer and participant) and took field notes. Interviews occurred between May-July 2014. She had never met participants, prior to conducting the interviews and explained that her purpose was to obtain their opinions and suggestions for how to implement a tablet assisted smoking cessation intervention in their clinic. Participants completed a brief pencil-and-paper demographic questionnaire. Semi-structured interviews were audio-recorded and transcribed for analysis. Participants received $25.00 for the interview. The University of California San Francisco institutional review board, called the Committee on Human Research, approved the study (12–09115) and written informed consent was obtained from all study participants. Transcripts were not returned to participants for comments.

### Analysis

Descriptive statistics were used to present demographic characteristics of participants. Data consisted of the verbatim transcripts of the interviews. The investigator who conducted the interviews coded the transcripts using NVivo qualitative data analysis software. Coding categories were initially based on the TAM constructs and expanded as needed based on additional constructs suggested by the data. Inductive coding techniques and a constant comparative approach were used to code the qualitative data [24]. The unit of analysis was an identifiable segment of continuous speech or text unit. A new text unit occurred when a speaker paused after a statement or a new speaker initiated; these ranged from a phrase to several paragraphs. Using an iterative process, preliminary findings were reviewed by the research team and discussed until consensus was reached on emerging themes, illustrative quotes, and implications for implementation of the intervention. Three practicing physicians (one from each of the clinic sites), the project director, and the principal investigator who is a behavioral health practitioner in one of the clinics, reviewed the emerging themes and representative quotes throughout the coding process. Their input and the field notes informed the final coding scheme, illustrative quotes selected, and interpretation of the findings. Results were compared by clinic site and employee position (administrative staff, clinical staff, or primary care provider). Due to time limitations, participants did not have the opportunity to provide feedback on the findings.

## Results

Out of 51 potential participants, 35 completed interviews, 13 did not respond to contact attempts, and three refused to participate. Thirty-five interviews were conducted until thematic saturation was reached (i.e., no new themes emerged). By design, the sample was distributed evenly across clinic sites and employee position. Most respondents were women; the majority was age 40 or older (Table 1). About a third were non-Latino White, and the rest were ethnically diverse. The themes emerging from the interviews are described below.

**Table 1:** Sample characteristics of primary care clinic staff and providers (*N* = 35)

Characteristic	N (%)
Age in years	
21–29	6 (17)
30–39	7 (20)
40–49	11 (31.5)
50–60	11 (31.5)
Ethnicity	
African American	5 (14)
American Indian	1 (3)
Asian	8 (23)
Latino	9 (26)
Non-Latino White	12 (34)
Female	32 (91)
Role in Clinic	
Administrative staff	12 (34)
Clinical staff	10 (29)
Primary care provider	13 (37)
Clinic site	
Public hospital HIV primary care practice	12 (34)
Public hospital academic general medicine clinic	11 (32)
Academic internal medicine practice	12 (34)

### Perceived usefulness of tablets in primary care

Perceptions about the general usefulness of tablet technology in primary care focused on five themes: 1) it can be used for a variety of administrative, health screening, and health counseling purposes; 2) it offers confidential collection of patient information; and 3) it can occupy patients while waiting for appointments. Regarding the potential usefulness of the CF5As specifically, two themes emerged: 1) its potential for increasing the efficiency of clinicians’ behavioral counseling of patients and 2) it can serve as a cue to address patients’ smoking in the context of the visit.

#### Tablets can be used for a variety of patient registration, screening, and health counseling purposes

It was perceived that technology could facilitate two-way communication by automating collection of routine information from patients and delivering health information to patients. Administrative staff saw the value of the tablet in facilitating their specific job functions, e.g., checking in patients and verifying insurance coverage. A front office staff person commented:

*“Tablets could be used for many, many things. We could confirm addresses, collect copayments, send appointment reminders electronically. We could check patients in that way. I just think it’s very exciting and I hope it does well. I would love to see our patients be able to use technology more to communicate with us and for us to be able to help them and support them more with technology (ADMINST#017).”*

Clinical staff described the potential utility of the tablet for collecting information from patients in terms of performing required routine health and functional status assessments and collecting information on medications, medical histories, family histories, pain, risk of falls, and basic needs (e.g., food insecurity). For example, a clinical staff person remarked:

*“I know a lot of different clinics in the first visit they kind of give you a little packet of how active you are on a daily basis. I think that would be a good thing to have, like those simple screening questions, like how active are you? What do you do? Are you interested in doing something else? And then that would trigger, if they do have like knee pain, shoulder pain, you know, to kind of prompt them to do certain types of exercises (CLINICST#007).”*

Participants of all positions remarked on the value of the tablet for conducting behavioral counseling. In addition to smoking cessation efforts, other health behavior counseling applications that were viewed as suitable for a tablet-based approach included patient education (including video) on physical activity, weight management, nutrition, managing depressive symptoms, use of alcohol and other substances, consumption of sugary beverages, low-salt diets, cholesterol, and understanding food labels. Other applications they identified included providing patients with information on preventive health screenings, vaccinations, test results, medication adherence, chronic disease self-management, stress management, patient activation, and community resources (e.g., food banks).

#### Tablets offer confidential collection of patient information

A common belief was that sensitive screening questions, such as those about sexual behavior, depressive symptoms, or drug use, could be collected confidentially, making patients more likely to disclose such behaviors. Administrative and clinical staff mentioned these advantages more frequently than primary care providers. A health care staff person commented:

*“It’s pretty well suited for smoking, substance use, and to try and get what symptoms people have. I also think it would work for the depression stuff. I do think people tend to be more honest when they are doing it on the tablet. And I think as long as the patient knows, I am going to hand this to your provider and your provider is going to go over it (PCP#108).”*

#### Tablets can occupy patients while waiting for appointments

Compared to those working in the academic internal medicine practice, personnel from the two safety-net hospital clinics reported significant wait times for patient visits and more often voiced opinions that wait times could be better utilized if the tablet was used to deliver health education to patients. For example, a clinician commented:

*“Patients oftentimes do wait for us a long time, and that is such a waste. If a patient has to wait for me for half an hour or an hour because I’ve gotten behind, that time could be spent learning things, going over medicines, having them do some education, and they wouldn’t feel so much like they had waited or it was a waste of time, and neither would I (PCP#011).”*

#### CF5As can increase the efficiency of clinician smoking cessation counseling of patients

Primary care providers and clinical staff in particular felt that the CF5As could allow physicians more time to focus on the information collected by the tablet, such as barriers to cessation, rather than having to spend that time asking the questions about smoking behavior and readiness to quit. In the words of one provider:

*“I think first of all, it’s a great conversation starter, and it may be a time saver, in terms of being able to scan quickly and see what people think the barriers are, what would motivate them more (PCP#011).”*

#### CF5As can serve as a prompt to counsel patients on smoking cessation

Personnel of all positions acknowledged that the CF5As might promote clinicians’ smoking cessation counseling by merely drawing attention to it. In busy practices characterized by high patient volumes and patients with multiple comorbidities, clinicians indicated that the CF5As would prompt them to address patients’ smoking behaviors when otherwise, they might prioritize addressing other, more pressing patient needs. Even an administrative assistant noted:

*“I think the benefits will be at least raising awareness not just for the provider, but also the patient. Then it sets the stage for the discussion to take place, ‘Okay, so I understand that you completed the questionnaire. I would like to discuss that with you. I know we have a lot to discuss, but instead of pushing it to be issue number 30 now maybe we’ll push it to be one of the top five.’ It sets up the stage for either the provider or the patient to initiate the conversation and start to think about behavioral changes (ADMINST#106).”*

### Perceived ease/difficulty of use

Perceptions about the ease of use of tablet technology in primary care focused on the diverse types of patients within the practices who might have difficulty with technology. With respect to CF5As implementation, two themes emerged: 1) the degree of ease of CF5As implementation depends on the extent to which it can be integrated easily into the workflow of the clinic; and 2) high patient volume will make it difficult to implement.

#### Tablet technology will be difficult for some of our patients

Patient characteristics described by all personnel, regardless of position, that would make use of tablet technology more difficult included older age, immigrant status, limited English proficiency, low literacy, and limited experience using computer technology. One clinician stated:

*“I feel like our older immigrants, like older patients in general (would have problems using it). I had a visual of a couple of older Latino or Chinese patients. I’m like, ‘No, I can’t imagine.’ Maybe I’m wrong. Maybe I’m not giving them credit, but then I’m also thinking of some older white patients whose English is perfect and I can’t imagine them using it (PCP#110).”*

Another clinician commented on the diversity of the patient population in terms of their computer literacy, socioeconomic status, substance use issues, and age that might make use of tablets difficult. In her words:

*“I think there are the people who are not comfortable with technology and don’t have a lot of computer or phone literacy. Our clinic population is really heterogeneous, I mean there are plenty of people who have lost private insurance and are coming here, but then there’s the homeless, marginalized substance using, mentally ill, very vulnerable population that hasn’t had access to that sort of thing. It’s older patients too. Patients over the age of 50, of which there are a lot (PCP#103).”*

#### CF5As ease of use depends on extent to which it can be integrated into clinic workflow

The overriding concern of respondents, regardless of position or site, was the potential impact of the CF5As on clinic workflow. The ease of use of CF5As was thought to depend on several key workflow-related factors: the time it would take patients to answer the questions; whether data could be synchronized with the EHR to obviate the need for data entry; the simplicity of the user interface; a touchscreen to minimize typing; the ability to identify patients that should receive the tablet at check-in; ensuring that the tablet did not interfere with rooming the patient and the visit; and a dedicated printer for the patient and provider handouts. Factors of concern to administrative staff were who would distribute and collect tablets and distribute patient and provider handouts; potential technical issues, e.g., poor connectivity; and what would happen if patients had no or short wait times. Providers and administrative staff especially, were worried about interference with workflow. For example, one clinician commented:

*“It needs to be done in such a way where it has as little impact on the work flow. Our flow and processes have changed so much so any little extra thing is going to potentially cause more of an issue even more so than before we went to the EMR (electronic medical record). That’s because we’re constantly being bombarded with requests throughout the day during visits. Any little bit is going to potentially derail the regular visit (PCP#221).”*

Another clinician commented:

*“I would be worried that it would slow down the check-in process for patients. My patients might come back to me maybe late then. If I am ready to see a patient right when they check in, I wouldn’t want them to spend ten minutes on a tablet, because then that might delay the start of my visit, which then makes me late for the next person (PCP015#).’*

#### CF5As will be difficult to implement due to high patient volume

Related to the clinic workflow, is the high-volume nature of these clinics. For example, an administrative staff person commented:

*“We have over 100 patient appointments per day, we have five tablets. That’s an issue right there. How do you determine who gets a tablet? How long do you let somebody sit with it? Whose responsibility is it to go out and take it away when somebody is done and collect it? It’s very busy here at our check in times. It will be hard for our clerks to add that into their role, just managing them (tablets) or like passing it to the next person or how will that work? They are not going to have the time to really do it effectively (ADMINST#17).”*

### Social norms, beliefs and attitudes

Two conflicting social norms emerged with respect to the implementation of health technology in primary care, the promise of technology and the burden of technology. Regarding the CF5As and smoking cessation specifically, we identified two social norms that could potentially affect adoption of the CF5As: the culture of the clinic with respect to the importance of smoking cessation counseling; and a prevailing sense that the CF5As would be viewed as just “one more thing to do” in the context of heavy workloads.

#### The promise of technology

Some believed that use of technology in primary care could increase workflow efficiency. In the words of an administrative assistant:

*“It’s better than paper. It’s electronic so it’s easier than trying to keep track of papers or giving the patient a paper, go to the waiting room, fill it out, bring it back. This is something that is being tabulated into an electronic thing that can be downloaded, and for most people it’s user friendly and so it’s better. If I was a patient, I would rather answer the questions in a tablet rather than writing something in (ADMINST#106).”*

Another clinical staff person remarked on the ubiquitous nature of technology, such that most patients would probably be familiar with and expect its use in primary care. She commented:

*“We’re in the 21*^*st*^*Century, I’m sure everybody is familiar with touchscreen gadgets in some way, if they go to the airport and check in with kiosks. If those are convenient for the airport, why can’t that be convenient for us, with the clientele we have? Because we have over 5,000 patients and every day we have almost 100 people check in, check out. It could help us move patients quicker, I think, with the technology (CLINICST#106).”*

#### The burden of technology

In contrast, clinical staff and clinicians commented on the burden of technology associated with EHR implementation and associated increases in reporting and accountability. Some providers viewed technology and EHRs as being “cumbersome,” and interfering with the physician-patient relationship due to having to enter information on computers during visits. Providers and clinical staff talked about the burden of changing health care system regulations that now require consistent recording of several clinical screening items in the EHR to fulfill meaningful use criteria. For example, one provider stated:

*“There is a whole list of 10 or 12 items that we are required to check and that takes time away from being able to actually do health care and counseling (PCP#012).”*

She referred to such requirements as “meaningless use,” indicating that the load was getting worse. In the words of another clinician:

*“The electronic medical records and the computer demand more attention than the patients because I frequently spend more visit time making the computer spit out a prescription or order a lab. So that’s time taken away from interaction with the patient. The patients arriving late, that kind of thing, you know bigger panels. And then all the demands to get funded we have to do certain tasks, like check boxes that may or may not be relevant to patient care, but if we don’t do them we don’t get paid (PCP#012).”*

#### Culture of the clinic with respect to the importance of smoking cessation counseling in the context of patients’ competing health needs

The extent to which clinic personnel felt that smoking cessation counseling was important varied by clinic site and was dependent to some extent on the existence of special quality initiatives focused on smoking cessation counseling. For example, a clinician from the academic primary care clinic commented:

*“Oh I think the norm is that it’s important (smoking cessation counseling) and actually doctors feel that they’re good at it. I don’t know if that’s true but we’ve had a lot of smoking cessation education in the last five to ten years, the 5As, and it’s been such a big public health issue. So I think doctors think they’re very good at it and they had the proper amount of training and so I think it’s a positive thing (PCP#214).”*

In two of the three sites, special initiatives were drawing attention to smoking cessation counseling. In the HIV clinic, because improving cessation rates in the practice had been the focus of a quality improvement initiative, one provider described a noticeable change as follows:

*“Some people knew the status of their patient. They might counsel them, but it was not being documented as well. But now that we are trying to do a smoking cessation initiative, it’s changed in that we are increasing the providers speaking with the patient as far as counseling. We are seeing the HAs (health assistants) enter that data into the EHR so that we can capture the data. There is actually an increased awareness because it’s being discussed more (PCP#106).”*

Generally, in the absence of such initiatives, smoking cessation counseling was viewed as low priority by health care staff and clinicians because it was time consuming and took time away from patients’ competing and more urgent health care needs. For example, a clinical staff person commented:

*“I don’t get any sense from the staff if it’s high priority to them or not. I get the sense that they ask these questions (whether patients smoke) because as part of check-in it’s one thing to check-off. We might get more rise out of staff if it’s like her blood sugar was just terrible and they ended up in the hospital because of the blood sugar. So, I feel everyone’s kind of neutral about it, but if they’re asked to spring into action they’re gonna do it (CLINICST#203).”*

Similarly, a clinician commented:

*“And the problem with something like smoking cessation or exercise is that it’s not one of those things that if you don’t solve it today, that person’s gonna be in a hospital tomorrow. And there are enough of those things in our patient population. Our patients are really, really sick, and although smoking cessation is incredibly important to many of the chronic illnesses that they have, if they’re coming in and they can’t breathe, you have to take care of that fact that they can’t breathe at that moment first before you can quite get to the smoking cessation (PCP#11).”*

#### Prevailing sense that the CF5As would be viewed as just “one more thing to do”

Across all of the clinics, perceptions of stressful workloads contributed to a view of the CF5As intervention as “just one more thing” to manage in an already overloaded clinic. A prevailing belief was that staff members were overburdened and constantly being asked to do more. Being asked to do more was viewed by one provider as being bad for the patient. Most providers indicated that although they recognized the importance of smoking cessation counseling, their ability to provide it was limited by the need to address urgent health issues during time-constrained visits. In the words of one clinician:

***“****I fear that right now people are seeing it as yet another thing to do on top of all of their work, and with everybody working as hard as they can, I don’t think that that’s sustainable (PCP#219).”*

### Facilitating conditions

One dominant theme emerged with respect to beliefs about the skills that would be needed to implement technology in the clinics. That theme had to do with the challenge of ensuring the level of effective communication among clinic personnel that would be needed to orient and train them and implement the necessary protocols related to use of the technology. Beliefs about infrastructure and resources viewed as influencing the potential success of the CF5As, specifically, included staffing configurations, the extent of available smoking cessation counseling resources and training, and lack of secure storage space for the tablets.

#### Challenge of ensuring the level of effective communication needed among clinic personnel

Opportunities to use technology, including the CF5As, were felt to depend largely on the capacity of the clinic to effectively communicate with providers and staff about its use. At all of the sites, administrative and health care staff and clinicians stated that effective communication among care teams would be necessary to ensure appropriate adoption of new technology and the CF5As protocol. In the public hospital general medicine clinic in particular, disseminating the information about CF5As to a large number of part-time house staff (i.e., residents and fellows) was viewed as particularly challenging. In the words of one provider:

*“The challenge is communicating it to all of our providers. We have 83 part-time providers in our clinic, and they are all there, nearly all of them, only one-half day a week. So I don’t know what the most feasible way would be to get the word out. What we rely on a lot is email. We are just starting to have bimonthly or quarterly provider meetings, which are mostly the faculty and the NPs, not the 50 residents, ‘cause they’re never there at the same time (PCP#014).”*

#### Staffing configurations

Two staffing issues surfaced that could facilitate or hinder implementation of the CF5As. One was the belief held by administrative staff that limited staffing would make CF5As implementation difficult and would require additional dedicated staff. In the words of one administrative staff person:

*“I think we are really understaffed in our clinic and probably every clinic says that. It is, it seems quite significant in our case and there is no one role, discipline who has the bandwidth for it the way things are structured now (ADMINST#105).”*

Almost all of the administrative staff mentioned that it would be helpful to have volunteers or study personnel assume responsibility for handing out and collecting the tablets from patients in the waiting room.

Clinicians, on the other hand, saw the potential for expanding the role of ancillary staff to include smoking cessation counseling. Clinicians from all the clinics commented on the possibility of engaging ancillary staff in the CF5As project. For example, one clinician commented:

*“Now with the reorganization of our clinic into these four panels, each panel has a nurse and a social worker assigned, and so the nurse and social worker have the potential to do a lot more I think now. I think one of the things we’re looking at for the panel is having the medical assistant or the health assistant take on more of a role. So, as they’re checking patients in, doing their vitals, that’s potentially an opportunity to ask about smoking (PCP#103).”*

In another of the clinics, a clinician indicated:

*“Yeah, but I think that what might make more sense is to have an extra MA (medical assistant) for each half day of clinic dedicated to smoking so that they’re not also getting pressure to check in patients. If my patient comes in and she knows my MA very well, my MA would be a better person to counsel on smoking cessation, but that float could come in and take my MA’s check-in duties while my MA is counseling (PCP#219).”*

#### Extent of smoking cessation counseling training and resources

The availability or lack of smoking cessation counseling training and resources was a dominant theme across many of the interviews, especially for health care staff and clinicians. Most clinicians mentioned wanting more training on behavioral counseling and motivational interviewing and many could not describe the 5As. For example, one provider mentioned:

*“I don’t know how to come up with different strategies and really – I don’t know how much more effective is doing a dual treatment. I just don’t know, and I don’t know how to advise people. I’m like, ‘Choose a quit date.’ How much before that do you start the bupropion? I’d have to look it up. I mean, I could figure it out. I feel like it would take a whole visit to really counsel them, start the medication, advise them how to take it, how to do a quit date, strategies. I just don’t do that (PCP#10).”*

Clinicians in both of the public hospital sites mentioned the lack of other smoking cessation resources in the clinics. In one site, the inability to refer patients to a smoking cessation class that had existed previously was mentioned by nearly all of the clinicians. In the words of one of the providers:

*“I think some of the difficulties that we run into are things like, we haven’t had smoking cessation classes for six months, and certainly I think in smoking cessation that can really help, having personal support and groups and people talking through the different things that they have tried (PCP#011).”*

However, at one of the public hospital clinics, a clinician commented:

*“We have another resource at the wellness center. They do classes on smoking cessation, so I refer people to that, too. So sometimes I’ll focus a little bit more on the patches or bupropion, and then sometimes I’ll have someone I delegate the behavioral part of it to. It depends on how much time I have (PCP#015).”*

Other barriers cited included lack of information on health insurance coverage of smoking cessation aids and lack of simple patient handouts on cessation resources. According to clinicians, resources such as behavioral health professionals who could spend more time counseling patients, would allow providers to focus on other health priorities.

#### Lack of secure storage space for the tablets

Storage space for the tablets was viewed as a limited resource. A common perception was that without secure space, the potential for theft of the tablets was fairly high. This concern was voiced more frequently by administrative staff. For example, one administrative person stated:

*“The office is about six, seven people and sometimes it’s so crowded. We need like a space if we’re going to deal with the tablets we need a space and secured place because you know, the office, practically everything disappears and we don’t want to be responsible for that because it is hard to keep it (ADMINST#2).”*

### Suggestions for implementation of the CF5As

Administrative staff had a number of suggestions to facilitate CF5As implementation. They suggested having a volunteer to hand out and collect the tablets and assist patients in using the tablet and offering a class to instruct patients on use of the tablet. Also, they suggested that patients be scheduled to arrive early for appointments to complete the assessment in the waiting room, but pointed out that this would require identifying smokers ahead of time. Almost all participants suggested that simplicity guide the design of the tablet interface and handouts due to the limited time of providers as well as the lower literacy levels of some patients. Clinicians suggested that providing them with feedback on the percentage of smokers in their patient panel who had been counseled would be a motivator to use the CF5As handout to counsel patients. Some providers stated that being able to view the handout at the visit was an important feature because it could be applied directly. Providers indicated that providing next steps for cessation on the handout would prompt them to schedule a follow-up appointment to discuss this even if they did not have the time at the current visit. All participants, regardless of position, indicated that training and advising staff and clinicians on the CF5As in advance of its implementation was necessary, and that this could be accomplished via routine staff and provider meetings, pre-clinic conferences, and email.

### Summary of differences by clinic site and employee group

We noted a few overarching differences by site. Although important in all three sites, two barriers to implementation, difficulties in accommodating clinic flow and ineffective communication among staff and providers, were mentioned more often in the public hospital general medicine clinic than the other two sites. Additionally, although important in all sites, the lack of other smoking cessation resources as a barrier to successful implementation was mentioned more often by personnel from the two public health clinics than those from the academic general medicine clinic.

Regarding differences by employee group, compared to administrative and health care staff, primary care providers more often mentioned the CF5As as “just one more thing to do,” patients’ competing health priorities and low literacy, limited knowledge of the 5As approach to smoking cessation, and lack of other smoking cessation resources as potential barriers to implementation. Administrative staff members were less likely than employees from the other two groups to mention the availability of other smoking cessation resources and the ability to integrate the CF5As into the clinic workflow as potential facilitators of implementation, and more likely to mention limited staffing to hand out and collect the tablets, as barriers.

## Discussion

Applying the TAM model, this pre-implementation qualitative study sought to identify from the perspectives of primary care clinic personnel, factors that might affect the implementation of technology-assisted behavioral counseling in general, and a computer-facilitated 5As model, specifically, in primary care settings. In our study, the perceived usefulness of tablets in primary care focused on the ability to perform a variety of administrative and health behavior counseling functions, collect patient data in a confidential manner, and deliver patient education during appointment wait times. Tablets were viewed as potentially improving the efficiency of behavioral counseling and serving as a cue to action for clinicians to initiate such counseling. The ease or difficulty of use of the CF5As model was viewed as dependent largely on the extent to which its implementation could accommodate, and not interrupt, clinic workflow. Heavy patient volume and patient characteristics, such as low literacy, limited English-proficiency, older age, and limited computer experience were viewed as factors that could hinder successful implementation of the CF5As and similar technology-assisted behavioral counseling interventions. The most frequently cited social norms potentially influencing the implementation of technology-assisted behavioral counseling, including the CF5As, was the view of technology as both a burden in terms of increased reporting and a promising tool to streamline clinic functions. Social norms that might influence CF5As implementation included the priority a particular clinic placed on smoking cessation counseling relative to other patient health needs and concern about the CF5As as “one more thing” to do in an overburdened health care setting where burnout and staff turnover are common. Conditions that would need to be addressed to facilitate CF5As implementation, included limited staffing, the need for additional smoking cessation resources and training, and secure storage space for the tablets.

Similar to prior research, participants in our study viewed one of the key factors affecting implementation of technology as the extent to which it could be integrated in the clinic so as to minimize its impact on patient flow [25]. Glasgow, et al., describe a variety of options for integrating interactive behavior change technologies to provide support before, during and after primary care visits, such as ongoing assessment and monitoring of patients’ health behaviors between outpatient visits or arranging linkages to community or peer support [26]. They point out also that the issue of integration into primary care could be tackled by healthcare delivery organizations and larger systems to help prioritize and standardize approaches and develop reimbursement mechanisms.

Although they were important factors mentioned by all sites, the tendency for the public hospital sites to more frequently mention clinic work flow, communication between staff and providers, and lack of other smoking cessation counseling resources as potential barriers could reflect the more limited resources and more challenging patient case mix of the public hospital clinics compared to the academic internal medicine practice. These findings indicate that attention to differences in resource constraints across implementation sites are important factors to consider when introducing new technology in primary care.

Our findings demonstrated that perceived barriers and facilitators of technology implementation in primary care are influenced to a great degree by the respective job functions of clinic personnel. For example, our findings indicate that primary care providers tend to view the impact and potential burden of introducing the CF5As as falling disproportionately on them, due to their primary role in providing smoking cessation counseling. This was evidenced by their greater likelihood of mentioning the CF5As as “just one more thing to do,” patients’ competing health priorities and low literacy, limited knowledge of the 5As approach to smoking cessation, and lack of other smoking cessation resources as potential barriers to implementation, compared to administrative and health care staff. Consistent with this finding, administrative staff members were less likely to mention the availability of other smoking cessation resources and the ability to integrate the CF5As into the clinic workflow as potential facilitators, compared to primary care providers and health care staff, probably due to their being the least involved in patient behavioral counseling. Administrative staff members were more concerned than health care staff and primary care providers about the distribution and collection of the tablets in the waiting room, which would fall most likely under their purview. Finally, some of the primary care providers suggested the possibility of an expanded role for the health care staff in smoking cessation counseling. Thus, implementation studies of the adoption of technology in primary care need to pay close attention to the impact of changes in job functions, which could potentially introduce conflict and other unintended consequences.

As in other studies, [16] we found important barriers to smoking cessation counseling to be lack of resources and self-efficacy for providing counseling and the competing health priorities of patients. Even with the availability of smoking cessation telephone quit lines in every state, physician referrals to this resource remain low [17]. Our results demonstrate that additional physician education regarding available and effective smoking cessation resources and counseling strategies is needed to build self-efficacy for providing such counseling.

However, physician education alone is insufficient. Likewise, interventions that focus on technology-delivered interventions alone have met with limited success [27, 28]. Stange, et al., argue that the key to leveraging the powerful influence of the clinician and the primary care encounter for the purpose of behavioral counseling lies in engaging physician-extenders before, during and after the patient-physician encounter [9]. Clinicians do not need to perform all of the 5As functions that are relevant for any health behavior counseling [9, 29]. The CF5As was designed to support clinicians’ efforts to conduct behavioral counseling during primary care visits by transferring some of the functions of asking, advising and assisting smoking cessation efforts to an interactive tablet. Additionally, family and community resources can be integrated with pragmatic, brief promotion of healthy behaviors in primary care, using a systems approach in which they are engaged in the advising, assisting and arranging steps [9]. Systems approaches can create synergy between ancillary staff, primary care clinicians, health technology support, and community resources [29, 30]. For example, a carefully integrated system consisting of e-referrals by physicians, a smoking cessation website, and brief motivational email messages was effective in increasing 6-month cessation rates, compared to paper referral only [31]. Multi-pronged efforts that enhance the behavioral health counseling efforts of clinicians with the use of telephones, videos, the Internet, and other computer-assisted methods, can reduce the services that must be provided directly by clinical staff and often provide the greatest benefits among low-income populations [29].

Computer-facilitated screening has improved the detection of unhealthy behaviors in primary care because it serves to prompt clinical teams to perform these tasks [32]; however, consistent delivery of behavioral counseling has proven more difficult. For example, a Morbidity and Mortality Weekly Report indicated that although tobacco use screening occurred during the majority of adult outpatient visits during 2005–2008 (62.7 %), among patients identified as current tobacco users, only 20.9 % received tobacco cessation counseling and 7.6 % received tobacco cessation medication [33]. In 2010, almost 70 % of smokers indicated a desire to quit smoking [33]. About 70 % of smokers make a physician visit every year, providing important opportunities for clinicians and clinical staff to intervene [33]. In our study, primary care providers and staff acknowledged the utility of the CF5As as a prompt to perform smoking cessation counseling and also saw its potential to free up clinicians to focus on counseling, rather than on the collection of data about patients’ smoking behaviors and cessation efforts. It is time to apply technology to reduce the health and social burden of smoking, especially since we have evidence-based interventions for cessation.

## Conclusions

Our results indicate that primary care personnel recognize the potential of technology to support clinician-delivered counseling. However for the “promise of technology” to materialize in the context of busy primary care practices, implementation will need to carefully weigh the impact of its introduction on job functions, resource constraints, and prevailing attitudes about such technology. Primary care clinicians face a number of challenges counseling their patients on lifestyle behaviors in the context of acute and chronic health care visits. Systematic integration of technology-assisted screening of smoking and other health behaviors and adjuncts to clinician-delivered behavioral counseling offer great promise for improving the behavioral health of primary care patients [30] if such challenges can be successfully addressed. To be most effective, technology-assisted behavioral counseling interventions must be integrated into the clinic flow and complement and extend rather than replace the efforts of the primary care team [28].

### Ethics approval and consent to participate

The University of California San Francisco Committee on Human Research approved the study (approval #12-09115). Written informed consent was obtained from all study participants.

### Consent for publication

Not applicable.

### Availability of data and materials

Because of the small sample sizes (especially, by position or site) and identification of the academic institution by name, it would be fairly easy to unveil the identity of participants. Thus, the transcripts of the interviews are not publically available.
